# Are one’s attachment avoidance toward a particular person and his/her placement of this particular person in the attachment hierarchy inversely overlapping? Four bifactor-analysis studies

**DOI:** 10.1371/journal.pone.0244278

**Published:** 2021-01-04

**Authors:** Tomotaka Umemura, Aneta Siroňová, Lenka Lacinová, Emiko Taniguchi, Tatsuya Imai

**Affiliations:** 1 Graduate School of Humanities and Social Sciences, Hiroshima University, Higashi-Hiroshima, Hiroshima, Japan; 2 Institute for Research on Children Youth and Family, Masaryk University, Brno, Czech Republic; 3 Department of Communicology, University of Hawai‘i at Mānoa, Honolulu, Hawaii, United States of America; 4 Department of British and American Studies, Nanzan University, Nagoya, Aichi, Japan; Technion Israel Institute of Technology, ISRAEL

## Abstract

Do one’s hierarchical preference for attachment support from a particular person over other people (attachment hierarchy) and his/her discomfort with closeness and uneasiness about being dependent on that particular person (attachment avoidance) inversely overlap? These two constructs have been distinctly conceptualized. Attachment hierarchy has been regarded as a normative characteristic of attachment relationships, while attachment avoidance has been considered to reflect an individual difference of relationship quality. Employing bifactor analyses, we demonstrated a unidimensional general factor of these two concepts in four studies exploring Czech young adults’ relationships with mother, father, friends, and romantic partner (Study 1); U.S. young adults’ relationships with a romantic partner (Study 2); Czech adolescents’ relationships with mother, father, and friends (Study 3); and Japanese young adults’ relationships with mother, father, and romantic partner (Study 4). These convergent results provide the replicable and generalizable evidence that one’s attachment avoidance toward a particular person and her/his placement of that particular person in the attachment hierarchy are inversely overlapping.

## Introduction

The extent to which two attachment constructs, one’s placement of a particular person in the attachment hierarchy and his/her attachment avoidance toward that particular person, inversely overlap each other has been unclear. In attachment research, these two constructs have been distinctly conceptualized. “Attachment hierarchy” has been defined as people’s propensity to hierarchically organize their significant others from whom they prefer to seek attachment. This concept has been regarded as a normative (or universal) process of attachment relationships (see Table 1). For example, adults who are in a stable romantic relationship tend to seek attachment support from a romantic partner rather than from other attachment figures, such as friends.

**Table 1:** The placement of a particular person in the attachment hierarchy and attachment avoidance toward a particular person account by both normative characteristics and individual differences.

	Previous Conceptualization		Our Conceptualization	
	Normative characteristics	Individual differences	Normative characteristics	Individual differences
**Attachment Hierarchy**	☑	☐	☑	☑
**Attachment Avoidance**	☐	☑	☑	☑

On the other hand, “attachment avoidance” has been defined as an individually different characteristic reflecting the degree to which individuals feel discomfort with closeness and uneasy about being dependent on a particular attachment figure. Attachment avoidance toward a particular person focuses on “a quality of attachment relationship” with a particular figure (see Table 1). This study examined whether the placement of a particular person in the attachment hierarchy and attachment avoidance toward a particular person inversely overlap.

### The concept of attachment hierarchy

Bowlby [1] and Ainsworth [2] proposed that infants and young children prefer the primary caregiver over other caregivers. During infancy and childhood, the mother is usually the primary caregiver and the most preferred attachment figure, unlike other attachment figures, such as the father (e.g., [3]) and day-care providers [4]. This hierarchical preference of attachment figures is believed to be associated with evolutionary survival instincts and, therefore, a *normative* (or universal) phenomenon for all children.

Throughout development, new attachment figures emerge, particularly friends and, later, romantic partners. In late adolescence and young adulthood, when a reproductive behavioral system becomes salient, the romantic partner or a sexual partner usually becomes the most preferred attachment figure (see [2, 5–7]). This is also a *normative* (or universal) process across young individuals.

### The concept of attachment avoidance

Although every person develops a hierarchy of attachment figures as a *normative* phenomenon, Ainsworth and her colleagues proposed that infants develop *individually different* attachment patterns with their caregivers based on their interactions. They assessed infants’ attachment by observing children’s behavioral interactions with their mother in a laboratory procedure, called “the strange situation procedure,” and categorized them into three categories: secure, insecure-avoidant, and insecure-ambivalent [8]. Infants who were classified as secure sought comfort from their caregiver and, soon afterward, confidently explored the environment. In contrast, infants classified as insecure-avoidant avoided seeking comfort from their caregiver, while infants categorized as insecure-ambivalent sought comfort but found it difficult to explore the environment away from the mother. These patterns of attachment reflect *individual differences* in infants’ attachment relationships with their caregivers.

Individual differences in attachment relationships have also been investigated in adults. Inspired by Ainsworth et al.’s [8] work on infant attachment patterns, subsequent researchers developed questionnaire items for adult romantic relationships, and factor analysis has identified two dimensions of adult attachment toward a romantic partner: attachment anxiety and attachment avoidance ([9, 10], see [11], for review). Attachment anxiety toward a romantic partner was defined as worries about abandonment or unavailability of a romantic partner and feelings of insufficient love. Attachment avoidance toward a romantic partner is referred to as one’s discomfort with closeness and uneasiness about being dependent on a romantic partner while preferring emotional distance and self-reliance. These two dimensions of attachment styles have recently been applied to adult attachment toward the mother, father, and friends [12]. In essence, one way to understand adult attachment is that adults show *individual differences* in these two dimensions of attachment styles, attachment anxiety and attachment avoidance, toward different persons.

### The inverse overlap between attachment hierarchy and attachment avoidance

Although attachment hierarchy and attachment avoidance concepts have been developed quite independently, they may not be completely different; instead, they may conceptually overlap. That is, the core idea of attachment hierarchy is “who would you go to,” while the core idea of attachment avoidance is “who would you avoid going to.” They seem inversely overlapping.

Although the possibility of normative changes in attachment avoidance toward a particular person (e.g., a romantic partner, [13]) has been discussed in the literature, little evidence of this possibility has been offered, except for a few studies. A previous study [14] provided evidence that as a romantic relationship progresses, young adults’ placement of romantic partner in the attachment hierarchy becomes more important, their placement of friends become less important, and their placement of parents shows no consistent pattern of change. In parallel, as a romantic relationship progresses, the degree of an individual’s attachment avoidance toward a romantic partner decreases, the degree of attachment avoidance toward friends increases, and the one toward parents shows no consistent pattern of change (Study 1 of the present study used the same data as the study mentioned above, but the previous study involved longitudinal data analysis, while the present study analyzed the first wave of the same data with a cross-sectional analysis). These findings indicated that attachment avoidance toward a particular person changes not only due to the quality of relationships but also due to the progress of relationships. Hence, although attachment avoidance toward a particular person has been considered to assess an *individual difference* in attachment relationships, it also seems to capture a *normative* process of attachment relationships (see Table 1).

On the other hand, the placement of persons in the attachment hierarchy is also accounted for by *normative* and *individually different* characteristics of attachment relationships. Previous studies [15, 16] have modeled attachment avoidance toward a romantic partner as the independent variable and a romantic partner's placement in the attachment hierarchy as the dependent variable. These studies assumed that if one has a more avoidant (or less secure) attachment style with a target person, he/she places this target person to be less important in his/her hierarchy; in other words, the *individual difference* in attachment avoidance toward a target person influences the placement of this target person in the attachment hierarchy. If this is the case, the placement of a particular person in the attachment hierarchy is also *individually different* and *normative* (see Table 1). However, none of these previous studies have examined the possibility of overlap between the two concepts.

### Bifactor analysis

To examine the overlap between the two concepts, attachment avoidance toward a particular person and the placement of a particular person in the attachment hierarchy, this study employed bifactor analysis, a type of higher-order confirmatory factor analysis (CFA). Bifactor models typically examine whether measured items comprise a unidimensional general domain and/or multiple domain-specific subdomains. This analysis approach has been used for theory testing. For example, according to Erikson’s [17] theory of psychosocial development, healthy young people develop one united identity. Although previous research has examined two dimensions of identity synthesis and identity confusion separately, studies using a bifactor analysis revealed the existence of a unidimensional general factor that captures both domains (e.g., [18]). This study proposed that a unidimensional general factor underlies the two attachment concepts.

### The present study

This study aimed to conduct bifactor analyses to examine whether the two attachment concepts (the placement of a particular individual in the attachment hierarchy and attachment avoidance toward a particular person) are inversely overlapping constructs. To examine this research question, we used four secondary datasets comprising participants of different ages (adolescents in secondary school and young adults in college) and nationalities (the Czech Republic, the U.S.A., and Japan). Employing these diverse datasets enabled us to demonstrate the *replicability* and *generalizability* of our results.

Specifically, the present study used four secondary datasets, which had been originally gathered for different purposes (see [19] for Study 1, [20] for Study 2, [21] for Study 3, & [22] for study 4). All the studies obtained the data for the mother, father, friend, and romantic partner, except for Study 2, which focused only on romantic relationships, and therefore only romantic data were obtained. Study 3 excluded the data on romantic relationships because only 29 adolescents reported being in a romantic relationship.

#### Data analysis

**Fig 1:**
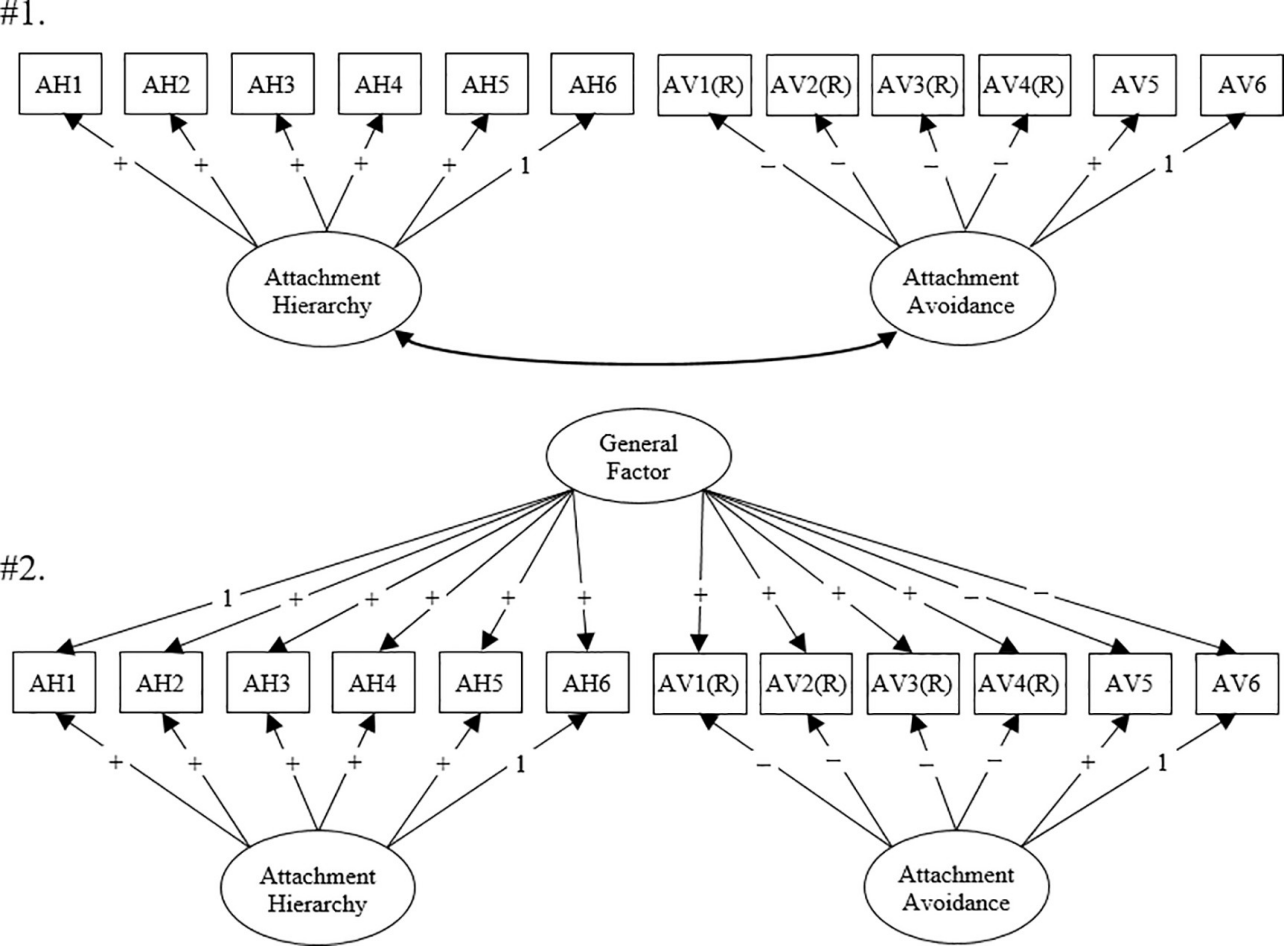
Data analyses in the present study. “AH” = Attachment Hierarchy. “AV” = Attachment Avoidance. “(R)” = reverse items. “1” indicates the fixed factor loading, and “+” and “-” signs indicate expected directions of factor loadings. Study 3 used a different Attachment Hierarchy measure, consisting of only three items.

We used the same two-step data analyses across the four studies. We ran the first-order CFA to ensure that all measured items generate two domain-specific latent variables (i.e., Attachment Hierarchy and Attachment Avoidance; see #1 in Fig 1). That is, the standardized factor loadings of measured items should not be small; for example, they should be greater than .40 in typical application. Additionally, the factor variance of latent variables should be significantly different from zero.

Subsequently, we tested bifactor CFA models (see #2 in Fig 1). Statistically, in a bifactor model, both general and domain-specific latent factors are directly regressed onto measured items. One general factor accounts for covariances among all measured items, while multiple domain-specific factors account for unique covariances among the measured items of each scale. In a typical parametrization of bifactor models, correlations between the general and domain-specific factors are set to zero to determine the variance that contributes uniquely to the general factor and the variances that contribute independently to domain-specific factors [23].

Because we expected to have high overlaps between Attachment Hierarchy and Attachment Avoidance, we hypothesized medium or large factor loadings for measured items on the general latent factor. We also hypothesized that factor loadings on the domain-specific factors in bifactor models would be much smaller in comparison to the factor loadings found in the previous first-order CFA results. In addition, we hypothesized that the domain-specific factor variances in bifactor models would not significantly differ from zero because the item variance would be used to build the factor variance of the general latent factor.

Finally, we also conducted the same two-step data analysis for the Attachment Hierarchy and Attachment Anxiety models to compare them with Attachment Hierarchy and Attachment Avoidance models. We expected that unlike the association between Attachment Hierarchy and Attachment Avoidance, the overlap between Attachment Hierarchy and Attachment Anxiety would be small. Therefore, we hypothesized to have medium or large factor loadings and significant factor variances in first-order CFA models. However, we also proposed that bifactor models would not generate the general factor with medium or large factor loadings or with a significant factor variance.

For all the two-step data analyses, we used the M*plus* program (version 7.11, [24]). In both the first-order and bifactor CFA analyses, items measuring Attachment Hierarchy were treated as categorical because the participants were asked to nominate the most significant person (Study 1 & Study 2) or to rank-order the first four significant persons (Study 3 & Study 4). On the other hand, items measuring Attachment Avoidance and Attachment Anxiety were treated as continuous because they were measured on a Likert-type scale.

## Study 1: Czech young adults’ attachment with mother, father, friends, and partner

### Method

#### Participants and procedure

Study 1 employed the data from Wave 1 of longitudinal research on Czech young adults aged 18 to 30 years (*M* = 21.57; *SD* = 1.53). The participants were recruited mostly from universities and other places, such as secondary schools, companies, employment offices, newspapers, local TV broadcasting, and online webpages (see [19]). They all completed online questionnaires. This study used 1,104 participants (79% females) who completed all attachment scales (out of 1,379 participants in total), of whom 633 participants were in a romantic relationship. The participants agreed on our informed consent form online, and we obtained the approval of this research project from the ethical committee of the Institute for Research on Children, Youth, and Family at Masaryk University.

#### Attachment hierarchy

We used a modified version of the WHOTO ([25]; the original version of WHOTO; [5]). This scale consists of 6 items measuring three conceptual subcategories: *secure base* (“Who is the person you would want to tell first if you achieved something good?” and “Who is the person you can always count on?”), *proximity seeking* (“Who is the person you don’t like to be away from?” and “Who is the person you most like to spend time with?”), and *safe haven* (“Who is the person you want to be with when you are feeling upset or down?” and “Who is the person you would count on for advice?”). The participants described the person(s) who best represented each item. A research assistant coded on a binary scale (0 = *no;* 1 = *yes*) whether the participants nominated the following attachment figures: the mother, the father, the friend, and/or the romantic partner. Internal consistencies were α = .72 (mother), α = .72 (father), α = .71 (friend), and α = .70 (romantic partner).

#### Attachment avoidance and attachment anxiety

The participants completed the 9-item Experiences in Close Relationships-Relational Structures scale (ECR-RS; [12]). Six items measure Attachment Avoidance (e.g., “I find it easy to depend on this person” and “I don’t feel comfortable opening up to this person”), and three items measure Attachment Anxiety (e.g., “I often worry that this person doesn't really care for me” and “I'm afraid that this person may abandon me”). The items are assessed on a 7-point scale (1 = *strongly disagree*; 7 *= strongly agree*). Internal consistencies for Attachment Avoidance were all good: α = .92 (mother), α = .89 (father), α = .89 (friend), and α = .86 (romantic partner). Internal consistencies for Attachment Anxiety were also all good: α = .73 (mother), α = .81 (father), α = .88 (friend), and α = .84 (romantic partner).

### Results

#### Descriptive analysis

Correlations between Attachment Hierarchy and Attachment Avoidance were medium: *r* = -.60 (mother), *r* = -.53 (father), *r* = -.43 (friend), and *r* = -.50 (romantic partner). Correlations between Attachment Hierarchy and Attachment Anxiety were small: *r* = -.24 (mother), *r* = -.27 (father), *r* = -.10 (friend), and *r* = -.26 (romantic partner). Table 2 summarizes the descriptive statistics for the sample in Study 1.

**Table 2:** Means (SDs) and correlation coefficients for the study variables in Czech young adults.

		*M*(*SD*)	*n*	1		2		3		4		5		6		7		8		9		10		11	
**Attachment Hierarchy**																									
**1**	**for mother**	.40(.30)	*n =* 1104																						
**2**	**for father**	.28(.28)	*n =* 1104	.59	***																				
**3**	**for friend**	.37(.31)	*n =* 1104	-.04		.05																			
**4**	**for partner**	.65(.30)	*n =* 633	-.01		.04		-.02																	
**Attachment Avoidance**																									
**5**	**for mother**	3.53(1.59)	*n =* 1104	-.60	***	-.22	***	.14	***	.01															
**6**	**for father**	4.38(1.51)	*n =* 1104	-.22	***	-.53	***	.03		.02		.22	***												
**7**	**for friend**	2.43(1.23)	*n =* 1104	-.07	*	-.07	*	-.43	***	.10	*	.17	***	.18	***										
**8**	**for partner**	2.01(.99)	*n =* 633	-.07	*	-.05		.10		-.50	***	.18	***	.13	**	.17	***								
**Attachment Anxiety**																									
**9**	**for mother**	1.71(1.11)	*n =* 1104	-.24	***	-.14	***	-.06	*	-.08	*	.31	***	.09	**	.11	***	.10	*						
**10**	**for father**	2.09(1.45)	*n =* 1103	-.13	***	-.27	***	-.04		-.04		.07	*	.38	***	.11	***	.08		.42	***				
**11**	**for friend**	2.58(1.54)	*n =* 1104	-.02		-.04		-.10	**	-.04		.11	***	.14	***	.30	***	.13	**	.25	***	.28	***		
**12**	**for partner**	2.69(1.50)	*n =* 663	-.10	*	-.08		-.05		-.26	***	.14	***	.12	**	.11	**	.28	***	.29	***	.29	***	.29	***

*Note*. Attachment Hierarchy ranges from 0 (lowest) to 1 (highest). Attachment Avoidance and Attachment Anxiety range from 1 (lowest) to 7 (highest).

*** *p* < .001.

** *p* < .01.

* *p* < .05.

#### First-order confirmatory factor analysis

We conducted eight first-order confirmatory factor analyses. Four models included latent factors of Attachment Hierarchy and Attachment Avoidance, and four models included Attachment Hierarchy and Attachment Anxiety. The four models in those two sets represent four relationships (mother, father, friend, and romantic partner). As expected, all eight first-order models had large levels of standardized factor loadings and significant levels of factor variances (see S1 Table, for all the information).

#### Bifactor analysis

Table 3 presents the bifactor models of Attachment Hierarchy and Attachment Avoidance. The standardized factor loadings of general factors, which comprised items from both Attachment Hierarchy and Attachment Avoidance, were either large or medium. The factor variance of the general factor was significant. These results provided evidence of a unidimensional general factor underlying Attachment Hierarchy and Attachment Avoidance, which supported our hypotheses.

**Table 3:** Bifactor analyses of attachment hierarchy and attachment avoidance in Czech young adults.

		Factor Loadings															
		Mother				Father				Friend				Partner			
Variables		b	(SE)		β	b	(SE)		β	b	(SE)		β	b	(SE)		β
**Attachment Hierarchy (AH)**	**AH1**	.48	(.08)***		.34	.59	(.08)***		.41	1.07	(.09)***		.67	.81	(.09)***		.61
	**AH2**	.58	(.07)***		.42	.69	(.07)***		.48	1.04	(.08)***		.65	.76	(.08)***		.57
	**AH3**	.46	(.10)***		.33	.69	(.11)***		.49	.60	(.08)***		.37	.60	(.07)***		.45
	**AH4**	.57	(.07)***		.41	.75	(.07)***		.53	.91	(.08)***		.56	.85	(.08)***		.64
	**AH5**	.71	(.08)***		.51	.75	(.07)***		.53	1.01	(.08)***		.63	.74	(.08)***		.56
	**AH6**	1.00	(.00)		.72	1.00	(.00)		.70	1.00	(.00)		.62	1.00	(.00)		.75
**Attachment Avoidance (AV)**	**AV1(R)**	.25	(.13)		.13	.14	(.10)		.07	.07	(.10)		.05	.78	(.45)		.26
	**AV2(R)**	-.56	(.06)***		-.26	-.30	(.05)		-.18	-.08	(.10)		-.05	1.20	(.65)		.38
	**AV3(R)**	-.64	(.06)***		-.30	-.30	(.05)		-.19	-.07	(.10)		-.04	1.13	(.62)		.33
	**AV4(R)**	.23	(.09)*		.11	.00	(.77)		.00	-.06	(.09)		-.03	.51	(.42)		.11
	**AV5**	1.17	(.08)***		.53	1.33	(.13)		.75	.92	(.17)***		.57	.84	(.44)		.22
	**AV6**	1.00	(.00)		.45	1.00	(.00)		.55	1.00	(.00)		.57	1.00	(.00)		.25
**General Factor (GF)**	**AH1**	1.00	(.00)		.54	1.00	(.00)		.46	1.00	(.00)		.40	1.00	(.00)		.43
	**AH2**	.84	(.07)***		.45	1.04	(.11)***		.47	.89	(.10)***		.36	.90	(.11)***		.39
	**AH3**	1.78	(.09)***		.63	1.09	(.15)***		.50	.1.28	(.12)***		.51	.1.09	(.12)***		.47
	**AH4**	1.24	(.09)***		.67	1.30	(.13)***		.59	1.20	(.12)***		.48	1.02	(.13)***		.44
	**AH5**	.89	(.08)***		.48	1.07	(.12)***		.49	.81	(.12)***		.32	.81	(.10)***		.35
	**AH6**	1.07	(.08)***		.57	1.25	(.12)***		.57	1.25	(.12)***		.50	1.07	(.11)***		.46
	**AV1(R)**	2.80	(.23)***		.88	3.92	(.41)***		.90	2.83	(.27)***		.84	1.69	(.22)***		.69
	**AV2(R)**	3.05	(.24)***		.87	3.14	(.31)***		.83	3.32	(.30)***		.89	2.13	(.28)***		.83
	**AV3(R)**	2.91	(.23)***		.81	2.79	(.28)***		.76	3.31	(.30)***		.84	2.20	(.30)***		.79
	**AV4(R)**	2.52	(.20)***		.71	3.39	(.36)***		.75	3.00	(.28)***		.73	2.56	(.33)***		.70
	**AV5**	-2.64	(.24)***		-.73	-2.23	(.29)***		-.53	-2.19	(.27)***		-.59	-2.06	(.26)***		-.67
	**AV6**	-2.45	(.23)***		-.67	-2.31	(.28)***		-.54	-2.31	(.29)***		-.58	-2.37	(.30)***		-.74
**Factor Variance**																	
** AH**		.51(.06)		***		.49(.05)		***		.39(.04)		***		.56(.06)		***	
** AV**		.79(.13)		***		1.16(.20)		***		.81(.20)		***		.12(.09)			
** GF**		.29(.04)		***		.21(.04)		***		.16(.03)		***		.19(.04)		***	
**Model fit indices**																	
** CFI**		.972				.960				.976				.933			
** RMSEA**		.042				.048				.041				.077			

*Note*. “AH” = Attachment Hierarchy. “AV” = Attachment Avoidance. “GF” = General Factor. “(R)” = reverse items.

*** *p* < .001.

** *p* < .01.

* *p* < .05.

One of the domain-specific factors, Attachment Avoidance, was problematic. Across all the relationship models, some or all standardized factor loadings were small. In the romantic relationship model, the factor variance of Attachment Avoidance was not significant.

Finally, bifactor models of Attachment Hierarchy and Attachment Anxiety were analyzed separately for different relationships. All models had errors, that is, the models for mothers, fathers, and romantic partners had negative general factor variances, and the model for friends had a negative value of the threshold of an observed variable.

## Study 2: The U.S. young adults’ attachment with a romantic partner

### Method

#### Participants and procedures

Study 2 recruited 294 heterosexual couples (588 individuals). This study used 581 individuals who completed all attachment scales. The data were collected from university students in the United States who received class bonus points for their participation (see [20]). The participants completed the questionnaire forms online. Ethnic characteristics of the participants were 54% Caucasian, 19% Hispanic, 12% Asian, 6% mixed, 5% African American, and 4% other ethnicities. Their age ranged from 16 to 43 (*M* = 21.10, *SD* = 2.76). The participants agreed on our informed consent form online, and the Institutional Review Board of University of Texas at Austin approved this research project (the protocol number: 2010-08-0027).

#### Attachment avoidance and attachment anxiety

The participants in Study 2 completed the ECR-RS, as in Study 1, but only for the romantic partner, since this research project originally focused exclusively on romantic relationships. Good internal consistency was obtained for attachment avoidance, α = .82, and attachment anxiety, α = .90.

#### Attachment hierarchy

The participants in Study 2 completed the WHOTO, as did participants in Study 1. The internal consistency for romantic partners was α = .73.

### Results

#### Descriptive analysis

The correlation between the Attachment Hierarchy and Attachment Avoidance was *r* = -.36, which was larger compared to the correlation between Attachment Hierarchy and Attachment Anxiety, *r* = -.19. This pattern was consistent with the results of Study 1. The descriptive statistics for the sample in Study 2 are presented in Table 4.

**Table 4:** Means (SDs) and correlation coefficients for the study variables in U.S. young adults.

		*M*(*SD*)	*n*	1		2	
**Attachment Hierarchy**							
**1**	**for partner**	.58(.30)	*n =* 581				
**Attachment Avoidance**							
**2**	**for partner**	1.75(.66)	*n =* 581	-.36	***		
**Attachment Anxiety**							
**3**	**for partner**	2.17(1.11)	*n =* 581	-.19	***	-.52	***

*Note*. Attachment Hierarchy ranges from 0 (lowest) to 1 (highest). Attachment Avoidance and Attachment Anxiety range from 1 (lowest) to 5 (highest).

*** *p* < .001.

** *p* < .01.

* *p* < .05.

#### First-order confirmatory factor analysis

Both first-order models for Attachment Hierarchy and Attachment Avoidance and Attachment Hierarchy and Attachment Anxiety had large or medium standardized factor loadings. The factor variances for all the domain-specific latent variables were significant (see S2 Table, for details).

#### Bifactor analysis

The results of the bifactor model for Attachment Hierarchy and Attachment Avoidance are presented in Table 5. The standardized factor loadings of general factors were all large or medium, except for a few items that had small scores. The factor variance of the general factor was also significant. These findings supported our hypotheses.

**Table 5:** Bifactor analysis of attachment hierarchy and attachment avoidance in American young adults.

		Factor Loadings		
		Partner		
Variables		b	(SE)	β
**Attachment Hierarchy (AH)**	**AH1**	.71	(.08)***	.53
	**AH2**	.83	(.08)***	.62
	**AH3**	.73	(.08)***	.54
	**AH4**	1.06	(.09)***	.78
	**AH5**	.98	(.07)***	.73
	**AH6**	1.00	(.00)	.74
**Attachment Avoidance (AV)**	**AV1(R)**	.52	(.24)*	.29
	**AV2(R)**	.54	(.23)*	.32
	**AV3(R)**	.57	(.26)*	.32
	**AV4(R)**	.57	(.24)*	.30
	**AV5**	1.89	(.70)**	.68
	**AV6**	1.00	(.00)	.38
**General Factor (GF)**	**AH1**	1.00	(.00)	.40
	**AH2**	.91	(.13)***	.36
	**AH3**	.98	(.14)***	.39
	**AH4**	.59	(.15)***	.20
	**AH5**	.57	(.13)**	.23
	**AH6**	.86	(.13)***	.34
	**AV1(R)**	1.40	(.18)***	.74
	**AV2(R)**	1.34	(.18)***	.75
	**AV3(R)**	1.52	(.20)***	.79
	**AV4(R)**	1.35	(.18)***	.66
	**AV5**	-2.11	(.37)***	-.71
	**AV6**	-1.67	(.27)***	-.60
**Factor Variance**				
** AH**		.55(.06)	***	
** AV**		.18(.08)	*	
** GF**		.16(.04)	***	
**Model fit**				
** CFI**		.957		
** RMSEA**		.056		

*Note*. “AH” = Attachment Hierarchy. “AV” = Attachment Avoidance. “AX” = Attachment Anxiety. “(R)” = reverse items.

*** *p* < .001.

** *p* < .01.

* *p* < .05.

However, consistent with Study 1, the domain-specific factor of Attachment Avoidance was problematic. The reversed items, which were supposed to have negative factor loadings, were positive. The factor variance was still significant, but the significance level was smaller compared to the one generated from the previous first-order model.

Finally, bifactor analyses were conducted for Attachment Hierarchy and Attachment Anxiety. However, the model terminated with errors (standard errors of the model parameter estimates could not be computed).

## Study 3: Czech adolescents’ attachment with mother, father, and friends

### Method

#### Participants and procedure

Study 3 employed adolescents' data gathered from students in three high schools in the Czech Republic (see [21], for details). This research utilized a two-year longitudinal design and measured Attachment Hierarchy and Attachment Avoidance simultaneously in one wave. This study employed 192 adolescents who completed all attachment scales (out of 215 participants in total). The participants' mean age was 14.47 years (*SD* = 2.04; ranging from 11 to 19 years), and 54% were females. Adolescents completed pen-and-paper questionnaires. Since only 29 adolescents reported being in a romantic relationship, we excluded the data on romantic relationships. Regarding research ethics, both adolescents and their parents signed the informed consent form. The ethical committee of the Institute for Research on Children, Youth, and Family at Masaryk University approved our research project.

#### Attachment hierarchy

Unlike Study 1 and Study 2, which employed the WHOTO scale and participants nominated only the best-matched attachment figure(s), Study 3 used the Important People Interview (IPI; [26]), asking participants to rank attachment figures in the first 4 places. Attachment figure(s) in the first place were assigned 4 points, attachment figure(s) in the second place were assigned 3 points, figures in the third place were given 2 points, figures in the fourth place were given 1 point, and finally, figure(s) who were not ranked in one of the four places were assigned 0 points.

The IPI was developed based on Bowlby’s [1] conceptualization of behavioral systems. Specifically, it consists of nine items measuring three subcategories: *attachment bond*, *support seeking*, and *affiliation*. This study used only three items of *attachment bond* (“To whom do you feel closest?;” “Imagine that you must fly across the country by yourself and stay by yourself for 2 weeks. Who would you miss the most?;” “Imagine you are walking by yourself. While crossing the street, you are suddenly hit by a car. The next thing you know, you are waking up in a hospital emergency room. Who do you want to see first?”). Internal consistencies were α = .82 (mother), α = .84 (father), and α = .79 (friends).

Previous studies [21, 26] have demonstrated psychometric properties of these three subcategories (*attachment bond*, *support seeking*, and *affiliation*) based on levels of attachment activation, *high*, *low*, and *no* degrees of distress, respectively. According to Bowlby’s [1] behavioral systems approach, interpersonal interactions during high levels of distress are critical for attachment because they are linked to survival. Because the IPI discriminates interpersonal interactions motivated by the activation of *attachment*, *instrumental support*, or *affiliative* systems, it has advanced the assessment of a person’s placement in the attachment hierarchy.

#### Attachment avoidance and attachment anxiety

The participants in Study 3 completed the ECR-RS, as did those in Study 1 and Study 2. Internal consistencies for Attachment Avoidance were all large: α = .90 (mother), α = .89 (father), and α = .83 (best friend). However, internal consistencies for Attachment Anxieity were medium to large: α = .59 (mother), α = .68 (father), and α = .86 (best friend).

### Results

#### Descriptive analysis

As in Study 1 and Study 2, the correlations between Attachment Hierarchy and Attachment Avoidance in Study 3 were medium: *r* = -.60 (mother), *r* = -.53 (father), and *r* = -.46 (friend). On the other hand, the correlations between Attachment Hierarchy and Attachment Anxiety in Study 3 were small: *r* = -.17 (mother), *r* = -.18 (father), and *r* = .05 (friend). The descriptive statistics for the sample in Study 3 are presented in Table 6.

**Table 6:** Means (SDs) and correlation coefficients for the study variables in Czech adolescents.

		*M*(*SD*)	*n*	1		2		3		4		5		6	7		8	
**Attachment Hierarchy**																		
** 1**	**for mother**	2.78(1.42)	*n =* 192															
** 2**	**for father**	2.10(1.51)	*n =* 192	.55	***													
** 3**	**for friend**	1.90(1.37)	*n =* 192	-.29	***	.37	***											
**Attachment Avoidance**																		
** 4**	**for mother**	3.14(1.42)	*n =* 192	-.60	***	-.26	***	.14										
** 5**	**for father**	3.75(1.51)	*n =* 192	-.26	**	-.53	***	.31	***	.41	***							
** 6**	**for friend**	2.47(1.15)	*n =* 192	.00		.11		-.46	***	.12		-.00						
**Attachment Anxiety**																		
** 7**	**for mother**	1.72(1.02)	*n =* 192	-.17	*	-.02		.02		.26	***	.12		.03				
** 8**	**for father**	1.85(1.15)	*n =* 192	-.08		-.18	*	.12		.18	*	.39	***	.04	.65	***		
** 9**	**for friend**	2.64(1.56)	*n =* 192	-.22	**	-.25	**	.05		.24	**	.29	***	.11	.43	***	.45	***

*Note*. Attachment Hierarchy ranges from 0 (lowest) to 4(highest). Attachment Avoidance and Attachment Anxiety range from 1 (lowest) to 7 (highest).

*** *p* < .001.

** *p* < .01.

* *p* < .05.

#### First-order confirmatory factor analysis

First-order analyses revealed that the models of two domain-specific latent factors of Attachment Hierarchy and Attachment Avoidance fit the data well. All the first-order models had large or medium standardized factor loadings and significant factor variances. In addition, we also found that the models of Attachment Hierarchy and Attachment Anxiety fit the data well, except for the model with friends (the friend model had an error with a negative value of a residual item variance). The detailed information is presented in S3 Table.

#### Bifactor analysis

Table 7 presents the bifactor models of Attachment Hierarchy and Attachment Avoidance. Consistent with Study 1 and Study 2, the general factor's standardized factor loadings were all large or medium. The factor variance of the general factor was also significant in all the models. Again, all these findings supported our hypotheses.

**Table 7:** Bifactor analyses of attachment hierarchy and attachment avoidance in Czech adolescents.

		Factor Loadings											
		Mother				Father				Friend			
Variables		b	(SE)		β	b	(SE)		β	b	(SE)		β
**Attachment Hierarchy (AH)**	**AH1**	1.06	(.13)***		.56	.77	(.09)***		.56	1.18	(.15)***		.65
	**AH2**	1.46	(.23)***		.77	.89	(.12)***		.64	1.53	(.25)***		.84
	**AH3**	1.00	(.00)		.53	1.00	(.00)		.72	1.00	(.00)		.55
**Attachment Avoidance (AV)**	**AV1(R)**	.78	(.92)		.28	-.12	(.27)		-.06	.13	(.15)		.14
	**AV2(R)**	-1.12	(.33)**		-.32	-.71	(.23)**		-.34	.04	(.14)		.04
	**AV3(R)**	-1.15	(.34)**		-.33	-.87	(.25)***		-.43	-.01	(.11)		-.01
	**AV4(R)**	.13	(.29)		.04	.01	(.29)		.01	.10	(.13)		.11
	**AV5**	.95	(.22)***		.26	1.28	(.34)***		.55	.36	(.27)		.37
	**AV6**	1.00	(.00)		.27	1.00	(.00)		.44	1.00	(.00)		.87
**General Factor (GF)**	**AH1**	1.00	(.00)		.64	1.00	(.00)		.62	1.00	(.00)		.39
	**AH2**	.99	(.07)***		.63	1.06	(.10)***		.66	1.12	(.21)***		.43
	**AH3**	.94	(.09)***		.60	.77	(.11)***		.48	.1.54	(.26)***		.60
	**AV1(R)**	1.99	(.25)***		.89	2.48	(.32)***		.90	2.91	(.55)***		.85
	**AV2(R)**	2.35	(.32)***		.83	2.33	(.34)***		.79	3.97	(.76)***		.91
	**AV3(R)**	2.07	(.31)***		.74	1.82	(.34)***		.64	3.31	(.64)***		.77
	**AV4(R)**	1.77	(.21)***		.73	2.25	(.29)***		.77	2.37	(.44)***		.64
	**AV5**	-2.08	(.34)***		-.70	-2.04	(.46)***		-.62	-1.62	(.47)**		-.44
	**AV6**	-2.05	(.34)***		-.68	-1.64	(.42)***		-.51	-2.01	(.70)**		-.46
**Factor Variance**													
** AH**		.28(.07)		***		.53(.10)		***		.30(.08)		***	
** AV**		.28(.22)				.76(.41)				2.18(1.84)			
** GF**		.41(.08)		***		.39(.07)		***		.15(.05)		**	
**Model fit**													
** CFI**		.998				.965				.965			
** RMSEA**		.021				.077				.072			

*Note*. “AH” = Attachment Hierarchy. “AV” = Attachment Avoidance. “GF” = General Factor. “(R)” = reverse items.

*** *p* < .001.

** *p* < .01.

* *p* < .05.

Consistent with Study 1 and Study 2, the domain-specific factor of Attachment Avoidance was again problematic. In each relationship model, some or all standardized factor loadings were small. The factor variances of Attachment Avoidance across all the relationship models were *not* significant.

Finally, bifactor analyses for Attachment Hierarchy and Attachment Anxiety were conducted. Like the results of Study 1 and Study 2, all models had errors. The models with mothers and fathers had a negative variance for the general factor. For the model with friends, standardized errors of model parameter estimates could not be computed.

## Study 4: Japanese young adults’ attachment with mother, father, friends, and romantic partner

### Method

#### Participants and procedure

Study 4 employed the data on young adults gathered from students at three Japanese universities (see [22], for details). We utilized a cross-sectional research design. This study employed 444 young adults who completed all attachment scales (out of 472 participants in total), and 172 young adults had a romantic partner. The participants' mean age was 20.36 years (*SD* = 1.29; ranging from 18 to 34 years), and 56% were females. After providing informed consent, the participants completed pen-and-paper questionnaires during a class. The research ethics committee of the Graduate School of Education at Hiroshima University approved this project.

#### Attachment hierarchy

Like Study 3, Study 4 used three items from the IPI [26]. Internal consistencies for Attachment Hierarchy were α = .65 (mother), α = .68 (father), α = .56 (friends), and α = .80 (romantic partner).

**Attachment avoidance and attachment anxiety** The participants in Study 4 completed the ECR-RS, as did those in the other Studies. Internal consistencies for Attachment Avoidance were all large: α = .88 (mother), α = .88 (father), α = .85 (best friend), and α = .85 (romantic partner). Similarly, internal consistencies for Attachment Anxieity were all large: α = .90 (mother), α = .93 (father), α = .92 (best friend), and α = .93 (romantic partner).

### Results

#### Descriptive analysis

As in the other studies, the correlations between Attachment Hierarchy and Attachment Avoidance in Study 4 were medium: *r* = -.55 (mother), *r* = -.53 (father), *r* = -.24 (friend), and *r* = -.58 (romantic partner). On the other hand, the correlations between Attachment Hierarchy and Attachment Anxiety in Study 4 were small: *r* = -.15 (mother), *r* = -.18 (father), *r* = .04 (friend), and *r* = -.15 (romantic partner). The descriptive statistics for the sample in Study 4 are also presented in Table 8.

**Table 8:** Means (SDs) and correlation coefficients for the study variables in Japanese young adults.

		*M*(*SD*)	*n*	1		2		3		4		5		6		7		8		9		10		11	
**Attachment Hierarchy**																									
** 1**	**for mother**	2.27(1.32)	*n =* 432																						
** 2**	**for father**	1.06(1.09)	*n =* 412	.43	***																				
** 3**	**for friend**	1.53(1.13)	*n =* 443	-.28	***	-.33	***																		
** 4**	**for partner**	2.11(1.45)	*n =* 175	-.07		-.10		-.30	***																
**Attachment Avoidance**																									
** 5**	**for mother**	3.00(1.34)	*n =* 437	-.55	***	-.28	***	.22	***	-.14															
** 6**	**for father**	3.80(1.44)	*n =* 420	-.18	***	-.53	***	.08		-.01		.44	***												
** 7**	**for friend**	2.59(1.07)	*n =* 437	-.05		.01		-.24	***	.01		.22	***	.20	***										
** 8**	**for partner**	2.73(1.14)	*n =* 220	-.15	*	-.03		.19	**	-.58	***	.33	***	.18	*	.41	***								
**Attachment Anxiety**																									
** 9**	**for mother**	2.03(1.23)	*n =* 436	-.15	**	-.14	**	.01		-.07		.30	***	.27	***	.26	***	.28	***						
** 10**	**for father**	2.07(1.25)	*n =* 419	-.06		-.18	***	.05		-.03		.19	**	.31	**	.28	***	.26	***	.67	***				
** 11**	**for friend**	2.91(1.52)	*n =* 437	-.01		-.08		.04		.07		.14	**	.22	***	.35	***	.15	*	.45	***	.43	***		
** 12**	**for partner**	3.38(1.83)	*n =* 220	-.04		-.17	*	.13	*	-.15		.15	*	.16	*	.12		.21	**	.28	***	.35	***	.42	***

*Note*. Attachment Hierarchy ranges from 0 (lowest) to 4 (highest). Attachment Avoidance and Attachment Anxiety range from 1 (lowest) to 7 (highest).

*** *p* < .001.

** *p* < .01.

* *p* < .05.

#### First-order confirmatory factor analysis

First-order analyses revealed that the models of two domain-specific latent factors of Attachment Hierarchy and Attachment Avoidance fit the data well. We also found that the models of Attachment Hierarchy and Attachment Anxiety fit the data well. All the first-order models had large or medium standardized factor loadings and significant factor variances (see S4 Table, for all the information).

#### Bifactor analysis

Table 9 presents the bifactor models of Attachment Hierarchy and Attachment Avoidance. All the models had a good model fit, except for the model with friends, in which the standard errors of parameters could not be estimated, and the residual covariance was not positive definite. Nonetheless, for the bifactor models with the mother, father, and romantic partner, the standardized factor loadings of the general factor were all large or medium. The factor variance of the general factor was also significant in all the models.

**Table 9:** Bifactor analyses of attachment hierarchy and attachment avoidance in Japanese young adults.

		Factor Loadings															
		Mother				Father				Friend				Partner^1^			
Variables		b	(SE)		β	b	(SE)		β	b	(SE)		β	b	(SE)		β
**Attachment Hierarchy (AH)**	**AH1**	.67	(.15)***		.35	.86	(.20)***		.50					.82	(.16)***		.48
	**AH2**	1.31	(.36)***		.68	.86	(.20)***		.49					1.20	(.28)***		.69
	**AH3**	1.00	(.00)		.52	1.00	(.00)		.58					1.00	(.00)		.58
**Attachment Avoidance (AV)**	**AV1(R)**	.44	(.25)		.24	2.06	(.99)*		.53					.53	(.43)		.21
	**AV2(R)**	.46	(.25)		.24	1.67	(.67)*		.43					.64	(.48)		.23
	**AV3(R)**	.07	(.14)		.04	-.14	(.29)		-.04					.93	(.61)		.35
	**AV4(R)**	-.05	(.10)		-.04	-.40	(.23)		-.11					.50	(.41)		.18
	**AV5**	.42	(.16)*		.23	.35	(.16)*		.10					1.26	(.80)		.40
	**AV6**	1.00	(.00)		.60	1.00	(.00)		.27					1.00	(.00)		.43
**General Factor (GF)**	**AH1**	1.00	(.00)		.61	1.00	(.00)		.60					1.00	(.00)		.59
	**AH2**	.71	(.08)***		.43	.81	(.09)***		.49					.99	(.13)***		.58
	**AH3**	.80	(.08)***		.49	.92	(.08)***		.55					.97	(.12)***		.57
	**AV1(R)**	2.49	(.21)***		.85	2.56	(.29)***		.83					1.72	(.26)***		.75
	**AV2(R)**	2.51	(.23)***		.82	2.30	(.27)***		.76					1.96	(.29)***		.76
	**AV3(R)**	2.42	(.17)***		.89	2.71	(.26)***		.87					2.04	(.30)***		.82
	**AV4(R)**	1.73	(.13)***		.73	2.28	(.22)***		.78					2.00	(.30)***		.79
	**AV5**	-1.32	(.17)***		-.46	-1.50	(.17)***		-.52					-1.74	(.36)***		-.59
	**AV6**	-2.02	(.21)***		-.75	-2.32	(.24)***		-.78					-1.39	(.24)***		-.64
**Factor Variance**																	
** AH**		.27(.09)		**		.33(.09)		***						.34(.10)		**	
** AV**		.95(.50)				.22(.13)								.30(.23)			
** GF**		.37(.05)		***		.36(.05)		***						.35(.08)		***	
**Model fit**																	
** CFI**		.967				.961								.965			
** RMSEA**		.056				.060								.077			

*Note*. “AH” = Attachment Hierarchy. “AV” = Attachment Avoidance. “GF” = General Factor. “(R)” = reverse items. ^1^To improve the model fit, we added correlations between AV1 and AV2 and between AV4 and AV5.

*** *p* < .001.

** *p* < .01.

* *p* < .05.

In addition, consistent with the other Studies, the domain-specific factor of Attachment Avoidance was again problematic. Some standardized factor loadings were small. The factor variances of Attachment Avoidance were *not* significant.

Finally, bifactor analyses for Attachment Hierarchy and Attachment Anxiety were conducted. Like the results of the other studies, all models had errors. All the models had a negative variance for the general factor, and standardized errors of model parameter estimates could not be computed.

## Discussion

The phenomena that individuals prefer to seek attachment support hierarchically from a particular person over other people (the attachment hierarchy concept) and that individuals feel discomfort with closeness and uneasiness about being dependent on a particular person (the attachment avoidance concept) may have an inversely overlapping meaning. The concept of attachment hierarchy has been considered to capture a normative process of attachment relationships. For example, adults in a stable romantic relationship prefer their romantic partner to fulfill their attachment needs over other attachment figures, such as friends and parents. On the other hand, the concept of attachment avoidance toward a particular person has been considered to capture an individually different quality of attachment relationships, reflecting an insecure (vs. secure) history of relationships. However, we proposed that the placement of a particular person in the attachment hierarchy may also capture an individually different quality of attachment relationships, and attachment avoidance toward a particular person may also capture a normative characteristic of attachment relationships. Accordingly, these two concepts of attachment could be inversely overlapping. To examine this possibility, this study employed bifactor analysis.

### Attachment hierarchy and attachment avoidance

As we hypothesized, our bifactor analyses from the four studies demonstrated that a unidimensional general factor underlies the two concepts: the placement of a particular person in the attachment hierarchy and attachment avoidance toward a particular person. Specifically, the general factor had medium to large standardized factor loadings and a significant factor variance across different relationships. These findings were consistent across four different samples in three counties (the Czech Republic, the United States, & Japan), two developmental groups (adolescents & young adults), and four relationships (mother, father, friend, & romantic partner). Therefore, the four studies provided convincing evidence of the *replicability* and *generalizability* of our findings.

In contrast, domain-specific factors sometimes had small factor loadings or medium/large ones but in unexpected directions. They also sometimes had a non-significant factor variance. These results indicate that items of the two concepts have overlapping covariance.

The placement of persons in one’s attachment hierarchy depends on both normative and individually different characteristics. On the one hand, regarding normative changes in one’s placement of particular persons in the attachment hierarchy, two lines of evidence have been reported. One is the developmental transfer of attachment figures from parents to peers (friends and romantic partners). Young adolescents are more likely to prefer parents over peers to seek attachment support, while late adolescents and young adults are more likely to prefer peers over parents [5, 27, 28]. Another evidence of normative changes is that an adult who engages in a long-term romantic relationship is likely to regard his/her romantic partner as a *more* important person in his/her attachment hierarchy, whereas an adult who has just started a romantic relationship is likely to regard a romantic partner as a *less* important person [14–16, 25, 26, 28]. These two phenomena are considered normative.

On the other hand, regarding individually different patterns of attachment hierarchy placement, there are two lines of evidence. Adolescents who have unsupportive parents transfer their attachment figures from parents to peers earlier compared to those who have supportive parents [26]. Their higher preference for friends and lower preference for parents were linked to emotional and behavioral problems [21, 26], suggesting that a premature transfer from parents to peers is a risk factor for adolescents’ adjustment [29]. Another evidence is that an individual who has an unsupportive romantic partner is likely to place the partner low in the attachment hierarchy; in contrast, if a romantic partner is supportive, regardless of the duration of the relationship, the partner is likely to be placed high in one’s attachment hierarchy [14–16]. Hence, we believe that a particular person's placement in the attachment hierarchy includes both normative and individually different components.

In a similar vein, attachment avoidance toward a particular person also changes depending on individual differences and normative characteristics of attachment relationships. For example, drawing upon our previous longitudinal study [14], suppose that one had a long-term supportive friend and recently started having a supportive romantic partner. This person would increase his/her attachment avoidance toward this friend as his/her romantic relationship progresses. This is a normative process unrelated to the quality of the attachment relationship with this friend. Hence, attachment avoidance toward a particular person is partly normative.

### Attachment hierarchy and attachment anxiety

We further tested the models of attachment anxiety toward a particular person and the placement of a particular person in the attachment hierarchy. All the bifactor models had some types of errors. For example, in some models, the general factor had a negative factor variance. These errors suggest that a particular person's placement in the attachment hierarchy and attachment anxiety toward a particular person do not overlap. On the other hand, all the first-order CFA models, except for one model (attachment with friends in Study 3), had medium or large standardized factor loadings and significant factor variances for the placement of a particular person in the attachment hierarchy and attachment anxiety toward a particular person. These findings suggest that these two attachment concepts are distinct.

## Limitations

This study depended solely on bifactor data analyses. More evidence is needed using experimental and qualitative research designs.

Our research mostly utilized sample sizes with sufficient statistical power. The minimum number of respondents per one parameter is considered five (e.g., [30], for review), and 42 parameters were involved in our most complex models, which required *N* >210. All sample sizes exceeded this minimum number, except for Study 3 (*N* = 192) and one model in Study 4 (*N* = 172 participants in a romantic relationship). However, regardless of the sample sizes, all our studies (except for one model regarding friends in Study 4) yielded the same pattern of results, which indicated that our findings were not due to statistical power.

Study 4 did not successfully estimate the bifactor model with friends, although it had a sufficient sample size. One reason could be that friends' placement in the attachment hierarchy did not have a good internal consistency. The first question of the IPI, “To whom do you feel closest?,” does not measure young adults’ attachment preferences during the moment of distress, whereas the other questions of the IPI specifically measure attachment preferences during distress moments. In Japanese culture, “closest” friends may not necessarily be “attachment” friends (see [22], for more details).

## Conclusion

The extent to which a particular person's placement in the attachment hierarchy and attachment avoidance toward a particular person overlap has been unclear. This study has demonstrated that these two concepts are inversely overlapping. This study contributes to the literature by suggesting that a particular person's placement in the attachment hierarchy and attachment avoidance toward a particular person are both normative and individually different.

According to Bowlby [31], “in the working model of the world that anyone builds, a key feature is his notion of who his attachment figures are, where they may be found, and how they may be expected to respond (p. 203).” That is, individuals spontaneously evaluate the information about these elements to obtain their emotional security. However, previous studies have examined one’s hierarchical preferences for attachment figures and his/her evaluation of relationship quality separately. Our study suggested that to understand better the complex nature of internal working models of attachment, future studies need to incorporate both elements.

We further suggest using a longitudinal research design for a more comprehensive understanding of attachment internal working models. Bowlby [1] proposed that attachment working models require modifications corresponding to one’s organism and environment:

…two working models each individual must have are referred to respectively as his environmental model and his organismic model. To be useful both working models must be kept up-to-date. As a rule this requires only a continuous feeding in of small modifications, usually a process so gradual that it is hardly noticeable. Occasionally, however, some major change in environment or in organism occurs: we get married, have a baby, or receive promotion at work; or, less happily, someone close to us departs or dies, a limb is lost, or sight fails. At those times radical changes of model are called for (p. 82).

Some organismic changes are more normative (e.g., developmental maturity), while some environmental changes are more individually unique (e.g., family economy). We propose that these organismic and environmental changes influence all the elements of attachment working models in qualitatively different manners. For example, developmental maturity may have a stronger influence on one’s hierarchical changes of attachment preferences, while the family economy may have a stronger effect on one’s relationship quality with his/her attachment figures. A more comprehensive understanding of attachment in adolescence and adulthood is needed.

## Supporting information

S1 Table Frist-order confirmatory factor analyses of Attachment Hierarchy and Attachment Avoidance (top) and of Attachment Hierarchy and Attachment Anxiety (bottom) in Czech young adults.
(DOCX)

S2 Table Frist-order confirmatory factor analyses of Attachment Hierarchy and Attachment Avoidance (top) and of Attachment Hierarchy and Attachment Anxiety (bottom) in American young adults.
(DOCX)

S3 Table Frist-order confirmatory factor analyses of Attachment Hierarchy and Attachment Avoidance (top) and of Attachment Hierarchy and Attachment Anxiety (bottom) in Czech adolescents.
(DOCX)

S4 Table Frist-order confirmatory factor analyses of Attachment Hierarchy and Attachment Avoidance (top) and of Attachment Hierarchy and Attachment Anxiety (bottom) in Japanese young adults.
(DOCX)
