# Supplementary material for: Are one’s attachment avoidance toward a particular person and his/her placement of this particular person in the attachment hierarchy inversely overlapping? Four bifactor-analysis studies
Source: PLoS One. 2021 Jan 4;16(1):e0244278. doi: 10.1371/journal.pone.0244278 (PMC7781391; doi:10.1371/journal.pone.0244278)
Supplement: S1 Table — Frist-order confirmatory factor analyses of Attachment Hierarchy and Attachment Avoidance (top) and of Attachment Hierarchy and Attachment Anxiety (bottom) in Czech young adults. (DOCX) [file pone.0244278.s001.docx]

**S1 Table. Frist-order confirmatory factor analyses of Attachment Hierarchy and Attachment Avoidance (top) and of Attachment Hierarchy and Attachment Anxiety (bottom) in Czech young adults.**

|  |  | **Factor Loadings** | | | | | | | | | | | | | | | | | | |
| --- | --- | --- | --- | --- | --- | --- | --- | --- | --- | --- | --- | --- | --- | --- | --- | --- | --- | --- | --- | --- |
|  |  | **Mother** | | | |  | **Father** | | | |  | **Friend** | | | |  | **Partner** | | | |
| **Variables** |  | **b** | **(SE)** | | **β** |  | **b** | **(SE)** | | **β** |  | **b** | **(SE)** | | **β** |  | **b** | **(SE)** | | **β** |
| **Attachment Hierarchy**  **(AH)** | **AH1** | .82 | (.05)^***^ | | .67 |  | .74 | (.05)^***^ | | .64 |  | .98 | (.05)^***^ | | .78 |  | .90 | (.06)^***^ | | .75 |
|  | **AH2** | .74 | (.04)^***^ | | .60 |  | .79 | (.04)^***^ | | .68 |  | .91 | (.05)^***^ | | .72 |  | .82 | (.06)^***^ | | .69 |
|  | **AH3** | .95 | (.05)^***^ | | .77 |  | .82 | (.07)^***^ | | .71 |  | .75 | (.05)^***^ | | .60 |  | .87 | (.05)^***^ | | .72 |
|  | **AH4** | 1.02 | (.04)^***^ | | .83 |  | .95 | (.04)^***^ | | .82 |  | .94 | (.05)^***^ | | .75 |  | .93 | (.05)^***^ | | .77 |
|  | **AH5** | .82 | (.04)^***^ | | .66 |  | .83 | (.04)^***^ | | .71 |  | .88 | (.05)^***^ | | .70 |  | .76 | (.06)^***^ | | .64 |
|  | **AH6** | 1.00 | (.00) | | .81 |  | 1.00 | (.00) | | .86 |  | 1.00 | (.00) | | .79 |  | 1.00 | (.00) | | .84 |
|  |  |  |  | |  |  |  |  | |  |  |  |  | |  |  |  |  | |  |
| **Attachment Avoidance**  **(AV)** | **AV1(R)** | -1.05 | (.07)^***^ | | -.85 |  | -1.45 | (.11)^***^ | | -.85 |  | -.94 | (.05)^***^ | | -.74 |  | -.81 | (.05)^***^ | | -.72 |
|  | **AV2(R)** | -1.22 | (.07)^***^ | | -.90 |  | -1.28 | (.09)^***^ | | -.86 |  | -1.17 | (.05)^***^ | | -.90 |  | -1.02 | (.06)^***^ | | -.85 |
|  | **AV3(R)** | -1.17 | (.07)^***^ | | -.85 |  | -1.14 | (.08)^***^ | | -.80 |  | -1.21 | (.05)^***^ | | -.86 |  | -1.06 | (.07)^***^ | | -.82 |
|  | **AV4(R)** | -.94 | (.07)^***^ | | -.68 |  | -1.30 | (.10)^***^ | | -.74 |  | -1.18 | (.06)^***^ | | -.70 |  | -1.22 | (.09)^***^ | | -.72 |
|  | **AV5** | 1.08 | (.06)^***^ | | .77 |  | .98 | (.05)^***^ | | .60 |  | .94 | (.04)^***^ | | .63 |  | .88 | (.05)^***^ | | .61 |
|  | **AV6** | 1.00 | (.00) | | .71 |  | 1.00 | (.00) | | .60 |  | 1.00 | (.00) | | .66 |  | 1.00 | (.00) | | .67 |
| **Factor Variance** |  |  | | | |  |  | | | |  |  | | | |  |  | | | |
| **AH** |  | .66(.04) | | ^***^ | |  | .74(.04) | | ^***^ | |  | .63(.04) | | ^***^ | |  | .70(.04) | | ^***^ | |
| **AV** |  | 1.94(.23) | | ^***^ | |  | 1.38(.19) | | ^***^ | |  | .95(.09) | | ^***^ | |  | .86(.10) | | ^***^ | |
| **Model fit** |  |  | | | |  |  | | | |  |  | | | |  |  | | | |
| **CFI** |  | .928 | | | |  | .930 | | | |  | .975 | | | |  | .922 | | | |
| **RMSEA** |  | .060 | | | |  | .056 | | | |  | .036 | | | |  | .074 | | | |

|  |  | **Factor Loadings** | | | | | | | | | | | | | | | | | | |
| --- | --- | --- | --- | --- | --- | --- | --- | --- | --- | --- | --- | --- | --- | --- | --- | --- | --- | --- | --- | --- |
|  |  | **Mother** | | | |  | **Father** | | | |  | **Friend** | | | |  | **Partner** | | | |
| **Variables** |  | **b** | **(SE)** | | **β** |  | **b** | **(SE)** | | **β** |  | **b** | **(SE)** | | **β** |  | **b** | **(SE)** | | **β** |
| **Attachment Hierarchy**  **(AH)** | **AH1** | .71 | (.04)^***^ | | .64 |  | .67 | (.05)^***^ | | .60 |  | .98 | (.05)^***^ | | .78 |  | .86 | (.06)^***^ | | .75 |
|  | **AH2** | .67 | (.04)^***^ | | .60 |  | .74 | (.04)^***^ | | .66 |  | .91 | (.05)^***^ | | .72 |  | .79 | (.06)^***^ | | .69 |
|  | **AH3** | .77 | (.05)^***^ | | .70 |  | .79 | (.06)^***^ | | .72 |  | .75 | (.05)^***^ | | .60 |  | .72 | (.05)^***^ | | .63 |
|  | **AH4** | .87 | (.04)^***^ | | .73 |  | .88 | (.04)^***^ | | .79 |  | .94 | (.05)^***^ | | .75 |  | .88 | (.06)^***^ | | .77 |
|  | **AH5** | .74 | (.04)^***^ | | .67 |  | .79 | (.04)^***^ | | .72 |  | .88 | (.05)^***^ | | .70 |  | .75 | (.06)^***^ | | .66 |
|  | **AH6** | 1.00 | (.00) | | .90 |  | 1.00 | (.00) | | .90 |  | 1.00 | (.00) | | .79 |  | 1.00 | (.00) | | .88 |
|  |  |  |  | |  |  |  |  | |  |  |  |  | |  |  |  |  | |  |
| **Attachment Anxiety**  **(AX)** | **AX1** | 1.42 | (.11)^***^ | | .93 |  | 1.38 | (.10)^***^ | | .93 |  | .68 | (.04)^***^ | | .74 |  | .81 | (.05)^***^ | | .78 |
|  | **AX2** | .58 | (.05)^***^ | | .38 |  | .78 | (.06)^***^ | | .57 |  | .82 | (.04)^***^ | | .80 |  | .88 | (.04)^***^ | | .81 |
|  | **AX3** | 1.00 | (.00) | | .81 |  | 1.00 | (.00) | | .81 |  | 1.00 | (.00) | | .95 |  | 1.00 | (.00) | | .92 |
| **Factor Variance** |  |  | | | |  |  | | | |  |  | | | |  |  | | | |
| **AH** |  | .82(.04) | | ^***^ | |  | .82(.04) | | ^***^ | |  | .63(.04) | | ^***^ | |  | .77(.05) | | ^***^ | |
| **AX** |  | .93(.09) | | ^***^ | |  | 1.55(.16) | | ^***^ | |  | .95(.09) | | ^***^ | |  | 2.45(.25) | | ^***^ | |
| **Model fit** |  |  | | | |  |  | | | |  |  | | | |  |  | | | |
| **CFI** |  | .954 | | | |  | .979 | | | |  | .973 | | | |  | .967 | | | |
| **RMSEA** |  | .062 | | | |  | .040 | | | |  | .042 | | | |  | .049 | | | |

*Note.* “AH” = Attachment Hierarchy. “AV” = Attachment Avoidance. “(R)” = reverse items.

*** *p* < .001. ** *p* < .01. * *p* < .05.
