# Supplementary material for: Are one’s attachment avoidance toward a particular person and his/her placement of this particular person in the attachment hierarchy inversely overlapping? Four bifactor-analysis studies
Source: PLoS One. 2021 Jan 4;16(1):e0244278. doi: 10.1371/journal.pone.0244278 (PMC7781391; doi:10.1371/journal.pone.0244278)
Supplement: S2 Table — Frist-order confirmatory factor analyses of Attachment Hierarchy and Attachment Avoidance (top) and of Attachment Hierarchy and Attachment Anxiety (bottom) in American young adults. (DOCX) [file pone.0244278.s002.docx]

**S2 Table. Frist-order confirmatory factor analyses of Attachment Hierarchy and Attachment Avoidance (top) and of Attachment Hierarchy and Attachment Anxiety (bottom) in American young adults.**

|  |  | **Factor Loadings** | | | |
| --- | --- | --- | --- | --- | --- |
|  |  | **Partner** | | | |
| **Variables** |  | **b** | **(SE)** | | **β** |
| **Attachment Hierarchy**  **(AH)** | **AH1** | .87 | (.07)^***^ | | .71 |
|  | **AH2** | .90 | (.07)^***^ | | .73 |
|  | **AH3** | .88 | (.06)^***^ | | .72 |
|  | **AH4** | .89 | (.07)^***^ | | .73 |
|  | **AH5** | .87 | (.06)^***^ | | .71 |
|  | **AH6** | 1.00 | (.00) | | .82 |
|  |  |  |  | |  |
| **Attachment Avoidance**  **(AV)** | **AV1(R)** | -1.25 | (.12)^***^ | | -.79 |
|  | **AV2(R)** | -1.19 | (.12)^***^ | | -.80 |
|  | **AV3(R)** | -1.36 | (.14)^***^ | | -.85 |
|  | **AV4(R)** | -1.21 | (.13)^***^ | | -.71 |
|  | **AV5** | 1.08 | (.09)^***^ | | .43 |
|  | **AV6** | 1.00 | (.00) | | .43 |
| **Factor Variance** |  |  | | | |
| **AH** |  | .67(.06) | | ^***^ | |
| **AV** |  | .23(.04) | | ^***^ | |
| **Model fit** |  |  | | | |
| **CFI** |  | .929 | | | |
| **RMSEA** |  | .065 | | | |

|  |  | **Factor Loadings** | | | |
| --- | --- | --- | --- | --- | --- |
|  |  | **Partner** | | | |
| **Variables** |  | **b** | **(SE)** | | **β** |
| **Attachment Hierarchy**  **(AH)** | **AH1** | .82 | (.07)^***^ | | .69 |
|  | **AH2** | .87 | (.06)^***^ | | .73 |
|  | **AH3** | .79 | (.06)^***^ | | .66 |
|  | **AH4** | .91 | (.06)^***^ | | .76 |
|  | **AH5** | .88 | (.06)^***^ | | .73 |
|  | **AH6** | 1.00 | (.00) | | .83 |
|  |  |  |  | |  |
| **Attachment Anxiety**  **(AX)** | **AX1** | 1.06 | (.08)^***^ | | .87 |
|  | **AX2** | 1.07 | (.08)^***^ | | .84 |
|  | **AX3** | 1.00 | (.00) | | .88 |
| **Factor Variance** |  |  | | | |
| **AH** |  | .69(.05) | | ^***^ | |
| **AX** |  | 1.02(.13) | | ^***^ | |
| **Model fit** |  |  | | | |
| **CFI** |  | .953 | | | |
| **RMSEA** |  | .057 | | | |

*Note.* “AH” = Attachment Hierarchy. “AV” = Attachment Avoidance. “AX” = Attachment Anxiety. “(R)” = reverse items. ^1^To improve the model fit, we added the correlation between AV5 and AV6.

*** *p* < .001. ** *p* < .01. * *p* < .05.
