# Supplementary material for: Are one’s attachment avoidance toward a particular person and his/her placement of this particular person in the attachment hierarchy inversely overlapping? Four bifactor-analysis studies
Source: PLoS One. 2021 Jan 4;16(1):e0244278. doi: 10.1371/journal.pone.0244278 (PMC7781391; doi:10.1371/journal.pone.0244278)
Supplement: S3 Table — Frist-order confirmatory factor analyses of Attachment Hierarchy and Attachment Avoidance (top) and of Attachment Hierarchy and Attachment Anxiety (bottom) in Czech adolescents. (DOCX) [file pone.0244278.s003.docx]

**S3 Table. Frist-order confirmatory factor analyses of Attachment Hierarchy and Attachment Avoidance (top) and of Attachment Hierarchy and Attachment Anxiety (bottom) in Czech adolescents.**

|  |  | **Factor Loadings** | | | | | | | | | | | | | | |
| --- | --- | --- | --- | --- | --- | --- | --- | --- | --- | --- | --- | --- | --- | --- | --- | --- |
|  |  | **Mother** | | | |  | **Father^1^** | | | |  | **Friend^2^** | | | |  |
| **Variables** |  | **b** | **(SE)** | | **β** |  | **b** | **(SE)** | | **β** |  | **b** | **(SE)** | | **β** |  |
| **Attachment Hierarchy**  **(AH)** | **AH1** | 1.07 | (.07)^***^ | | .88 |  | 1.05 | (.06)^***^ | | .85 |  | .63 | (.09)^***^ | | .62 |  |
|  | **AH2** | 1.14 | (.08)^***^ | | .94 |  | 1.17 | (.08)^***^ | | .94 |  | .74 | (.09)^***^ | | .73 |  |
|  | **AH3** | 1.00 | (.00) | | .82 |  | 1.00 | (.00) | | .81 |  | 1.00 | (.00) | | .98 |  |
|  |  |  |  | |  |  |  |  | |  |  |  |  | |  |  |
| **Attachment Avoidance**  **(AV)** | **AV1(R)** | -.87 | (.14)^***^ | | -.82 |  | -1.37 | (.25)^***^ | | -.88 |  | -1.64 | (.38)^***^ | | -.82 |  |
|  | **AV2(R)** | -1.14 | (.15)^***^ | | -.85 |  | -1.41 | (.26)^***^ | | -.86 |  | -2.34 | (.48)^***^ | | -.93 |  |
|  | **AV3(R)** | -1.00 | (.15)^***^ | | -.76 |  | -1.14 | (.21)^***^ | | -.72 |  | -1.96 | (.41)^***^ | | -.79 |  |
|  | **AV4(R)** | -.83 | (.13)^***^ | | -.72 |  | -1.21 | (.24)^***^ | | -.75 |  | -1.35 | (.31)^***^ | | -.63 |  |
|  | **AV5** | 1.01 | (.14)^***^ | | .72 |  | 1.25 | (.20)^***^ | | .69 |  | .89 | (.15)^***^ | | .41 |  |
|  | **AV6** | 1.00 | (.00) | | .70 |  | 1.00 | (.00) | | .56 |  | 1.00 | (.00) | | .39 |  |
| **Factor Variance** |  |  | | | |  |  | | | |  |  | | | |  |
| **AH** |  | .68(.08) | | ^***^ | |  | .65(.07) | | ^***^ | |  | .97(.12) | | ^***^ | |  |
| **AV** |  | 1.85(.54) | | ^**^ | |  | 1.27(.45) | | ^**^ | |  | .45(.19) | | ^*^ | |  |
| **Model fit** |  |  | | | |  |  | | | |  |  | | | |  |
| **CFI** |  | .972 | | | |  | .955 | | | |  | .977 | | | |  |
| **RMSEA** |  | .059 | | | |  | .075 | | | |  | .051 | | | |  |

|  |  | **Factor Loadings** | | | | | | | | | | | | | | |
| --- | --- | --- | --- | --- | --- | --- | --- | --- | --- | --- | --- | --- | --- | --- | --- | --- |
|  |  | **Mother** | | | |  | **Father** | | | |  | **Friend** | | | |  |
| **Variables** |  | **b** | **(SE)** | | **β** |  | **b** | **(SE)** | | **β** |  | **b** | **(SE)** | | **β** |  |
| **Attachment Hierarchy**  **(AH)** | **AH1** | 1.06 | (.07)^***^ | | .85 |  | .98 | (.05)^***^ | | .83 |  |  |  | |  |  |
|  | **AH2** | .1.22 | (.10)^***^ | | .98 |  | 1.10 | (.07)^***^ | | .93 |  |  |  | |  |  |
|  | **AH3** | 1.00 | (.00) | | .81 |  | 1.00 | (.00) | | .85 |  |  |  | |  |  |
|  |  |  |  | |  |  |  |  | |  |  |  |  | |  |  |
| **Attachment Anxiety**  **(AX)** | **AX1** | 1.08 | (.30)^***^ | | .60 |  | 2.16 | (.61)^***^ | | .88 |  |  |  | |  |  |
|  | **AX2** | .87 | (.24)^***^ | | .45 |  | 1.14 | (.28)^***^ | | .47 |  |  |  | |  |  |
|  | **AX3** | 1.00 | (.00) | | .71 |  | 1.00 | (.00) | | .57 |  |  |  | |  |  |
| **Factor Variance** |  |  | | | |  |  | | | |  |  | | | |  |
| **AH** |  | .65(.08) | | ^***^ | |  | .71(.07) | | ^***^ | |  |  | |  | |  |
| **AX** |  | .63(.20) | | ^**^ | |  | .43(.14) | | ^**^ | |  |  | |  | |  |
| **Model fit** |  |  | | | |  |  | | | |  |  | | | |  |
| **CFI** |  | .990 | | | |  | .967 | | | |  |  | | | |  |
| **RMSEA** |  | .070 | | | |  | .123 | | | |  |  | | | |  |

*Note.* “AH” = Attachment Hierarchy. “AV” = Attachment Avoidance. “(R)” = reverse items.

^1^To improve the model fit, we added correlations between AV5 and AV6. ^2^W added correlations between AH1 and AH2 and between AV5 and AV6.

*** *p* < .001. ** *p* < .01. * *p* < .05.
