# Supplementary material for: Are one’s attachment avoidance toward a particular person and his/her placement of this particular person in the attachment hierarchy inversely overlapping? Four bifactor-analysis studies
Source: PLoS One. 2021 Jan 4;16(1):e0244278. doi: 10.1371/journal.pone.0244278 (PMC7781391; doi:10.1371/journal.pone.0244278)
Supplement: S4 Table — Frist-order confirmatory factor analyses of Attachment Hierarchy and Attachment Avoidance (top) and of Attachment Hierarchy and Attachment Anxiety (bottom) in Japanese young adults. (DOCX) [file pone.0244278.s004.docx]

**S4 Table. Frist-order confirmatory factor analyses of Attachment Hierarchy and Attachment Avoidance (top) and of Attachment Hierarchy and Attachment Anxiety (bottom) in Japanese young adults.**

|  |  | **Factor Loadings** | | | | | | | | | | | | | | | | | | |
| --- | --- | --- | --- | --- | --- | --- | --- | --- | --- | --- | --- | --- | --- | --- | --- | --- | --- | --- | --- | --- |
|  |  | **Mother^1^** | | | |  | **Father** | | | |  | **Friend** | | | |  | **Partner^1^** | | | |
| **Variables** |  | **b** | **(SE)** | | **β** |  | **b** | **(SE)** | | **β** |  | **b** | **(SE)** | | **β** |  | **b** | **(SE)** | | **β** |
| **Attachment Hierarchy**  **(AH)** | **AH1** | 1.16 | (.10)^***^ | | .81 |  | 1.06 | (.08)^***^ | | .81 |  | .74 | (.11)^***^ | | .54 |  | .97 | (.08)^***^ | | .79 |
|  | **AH2** | .93 | (.09)^***^ | | .65 |  | .89 | (.08)^***^ | | .68 |  | .97 | (.14)^***^ | | .70 |  | 1.09 | (.10)^***^ | | .89 |
|  | **AH3** | 1.00 | (.00) | | .69 |  | 1.00 | (.00) | | .77 |  | 1.00 | (.00) | | .73 |  | 1.00 | (.00) | | .82 |
|  |  |  |  | |  |  |  |  | |  |  |  |  | |  |  |  |  | |  |
| **Attachment Avoidance**  **(AV)** | **AV1(R)** | -1.29 | (.11)^***^ | | -.82 |  | -1.13 | (.11)^***^ | | -.81 |  | -1.68 | (.14)^***^ | | -.81 |  | -1.56 | (.22)^***^ | | -.84 |
|  | **AV2(R)** | -1.30 | (.12)^***^ | | -.79 |  | -1.03 | (.10)^***^ | | -.74 |  | -1.80 | (.15)^***^ | | -.82 |  | -1.70 | (.24)^***^ | | -.82 |
|  | **AV3(R)** | -1.32 | (.09)^***^ | | -.90 |  | -1.24 | (.11)^***^ | | -.88 |  | -1.64 | (.14)^***^ | | -.86 |  | -1.67 | (.23)^***^ | | -.84 |
|  | **AV4(R)** | -.96 | (.07)^***^ | | -.75 |  | -1.03 | (.09)^***^ | | -.78 |  | -1.45 | (.12)^***^ | | -.77 |  | -1.63 | (.23)^***^ | | -.80 |
|  | **AV5** | .67 | (.07)^***^ | | .43 |  | .67 | (.06)^***^ | | .51 |  | 1.09 | (.10)^***^ | | .44 |  | 1.05 | (.18)^***^ | | .43 |
|  | **AV6** | 1.00 | (.00) | | .69 |  | 1.00 | (.00) | | .74 |  | 1.00 | (.00) | | .52 |  | 1.00 | (.00) | | .52 |
| **Factor Variance** |  |  | | | |  |  | | | |  |  | | | |  |  | | | |
| **AH** |  | .49(.06) | | ^***^ | |  | .58(.06) | | ^***^ | |  | .53(.10) | | ^***^ | |  | .67(.07) | | ^***^ | |
| **AV** |  | 1.28(.19) | | ^***^ | |  | 1.74(.28) | | ^***^ | |  | .46(.07) | | ^***^ | |  | .52(.14) | | ^***^ | |
| **Model fit** |  |  | | | |  |  | | | |  |  | | | |  |  | | | |
| **CFI** |  | .929 | | | |  | .928 | | | |  | .920 | | | |  | .906 | | | |
| **RMSEA** |  | .070 | | | |  | .068 | | | |  | .075 | | | |  | .097 | | | |

|  |  | **Factor Loadings** | | | | | | | | | | | | | | | | | | |
| --- | --- | --- | --- | --- | --- | --- | --- | --- | --- | --- | --- | --- | --- | --- | --- | --- | --- | --- | --- | --- |
|  |  | **Mother** | | | |  | **Father** | | | |  | **Friend** | | | |  | **Partner** | | | |
| **Variables** |  | **b** | **(SE)** | | **β** |  | **b** | **(SE)** | | **β** |  | **b** | **(SE)** | | **β** |  | **b** | **(SE)** | | **β** |
| **Attachment Hierarchy**  **(AH)** | **AH1** | .90 | (.10)^***^ | | .68 |  | .96 | (.10)^***^ | | .78 |  | .70 | (.11)^***^ | | .54 |  | .94 | (.07)^***^ | | .77 |
|  | **AH2** | .94 | (.11)^***^ | | .71 |  | .81 | (.08)^***^ | | .66 |  | .86 | (.16)^***^ | | .66 |  | 1.11 | (.10)^***^ | | .91 |
|  | **AH3** | 1.00 | (.00) | | .76 |  | 1.00 | (.00) | | .81 |  | 1.00 | (.00) | | .77 |  | 1.00 | (.00) | | .82 |
|  |  |  |  | |  |  |  |  | |  |  |  |  | |  |  |  |  | |  |
| **Attachment Anxiety**  **(AX)** | **AX1** | 1.01 | (.06)^***^ | | .81 |  | 1.12 | (.06)^***^ | | .92 |  | 1.23 | (.05)^***^ | | .92 |  | 1.26 | (.12)^***^ | | .96 |
|  | **AX2** | 1.24 | (.08)^***^ | | .97 |  | 1.15 | (.05)^***^ | | .95 |  | 1.23 | (.05)^***^ | | .96 |  | 1.30 | (.14)^***^ | | 1.00 |
|  | **AX3** | 1.00 | (.00) | | .80 |  | 1.00 | (.00) | | .83 |  | 1.00 | (.00) | | .80 |  | 1.00 | (.00) | | .77 |
| **Factor Variance** |  |  | | | |  |  | | | |  |  | | | |  |  | | | |
| **AH** |  | .58(.09) | | ^***^ | |  | .66(.08) | | ^***^ | |  | .59(.12) | | ^***^ | |  | .67(.08) | | ^***^ | |
| **AX** |  | 1.16(.13) | | ^***^ | |  | 1.23(.12) | | ^**^ | |  | 1.06(.20) | | ^***^ | |  | 2.21(.50) | | ^***^ | |
| **Model fit** |  |  | | | |  |  | | | |  |  | | | |  |  | | | |
| **CFI** |  | .981 | | | |  | .983 | | | |  | 1.000 | | | |  | .977 | | | |
| **RMSEA** |  | .049 | | | |  | .050 | | | |  | .000 | | | |  | .068 | | | |

*Note.* “AH” = Attachment Hierarchy. “AV” = Attachment Avoidance. “(R)” = reverse items.

^1^To improve the model fit, we added correlations between AV5 and AV6.

*** *p* < .001. ** *p* < .01. * *p* < .05.
